# Exercise-induced myocardial ischemia presenting as exercise intolerance after carbon monoxide intoxication and smoke inhalation Injury: case report

**DOI:** 10.1186/s12872-022-03019-4

**Published:** 2022-12-27

**Authors:** Yu-Mei Wang, Chao-Chun Huang, Kuan-Fu Liu, Chen-Liang Chou, Jen-Ting Lee, Su-Ying Hung, Po-Cheng Hsu

**Affiliations:** 1 Department of Physical Medicine and Rehabilitation, Taipei Veterans General Hospital, No. 201, Sec. 2, Shipai Rd., Beitou Dist., Taipei City 112, Taiwan (R.O.C.); 2 National Yang-Ming University, Taipei, Taiwan (R.O.C.); 3 Department of Physical Medicine and Rehabilitation, Far Eastern Memorial Hospital, New Taipei City, Taiwan (R.O.C.); 4 Department of Physical Medicine and Rehabilitation, West Garden Hospital, Taipei City, Taiwan; 5 Department of Physical Medicine and Rehabilitation, National Taiwan University Hospital, Bei-Hu Branch, Taipei City, Taiwan

**Keywords:** Exercise-induced myocardial ischemia, Exercise intolerance, Carbon monoxide intoxication, Smoke inhalation injury, Case report

## Abstract

**Background:**

Carbon monoxide intoxication and smoke inhalation injury can lead to severe disorders, and the current literature has elaborated on the importance of major cardiopulmonary impairment. Exercise intolerance has seldom been discussed, particular in patient with low cardiovascular risk.

**Case presentation:**

Two young male fire survivors who presented with exercise intolerance after CO intoxication and smoke inhalation injury. Both received bronchodilator and glucocorticoid therapy, high-flow oxygen therapy, and hyperbaric oxygen therapy for airway edema and CO intoxication during acute care. Serum carboxyhemoglobin levels improved after treatment (8.2–3.9% in Case A and 14.8–0.8% in Case B). However, subjective exercise intolerance was noted after discharge. Cardiopulmonary exercise testing revealed exercise-induced myocardial ischemia during peak exercise (significant ST-segment depression on exercise electrocardiogram). They were instructed to exercise with precaution by setting the intensity threshold according to the ischemic threshold. Their symptoms improved, and no cardiopulmonary events were reported in the 6-month follow-up.

**Conclusion:**

The present case report raised the attention that exercise intolerance after carbon monoxide intoxication and smoke inhalation injury in low cardiovascular risk population may be underestimated. Cardiopulmonary exercise testing help physician to discover exercise-induced myocardial ischemia and set up the cardiac rehabilitation program accordingly.

**Supplementary Information:**

The online version contains supplementary material available at 10.1186/s12872-022-03019-4.

## Background

Carbon monoxide intoxication and smoke inhalation injury remain clinical challenges in fire survivors. Apart from complex fatal organ damages, cardiopulmonary impairments such as acute airway obstruction, increased risk of pneumonia, myocardial stunning, left ventricular dysfunction, arrhythmia, and acute myocardial ischemia were often reported [1, 2]. However, exercise intolerance has seldom been discussed, especially in people with low cardiovascular risk. This case report aims to raise the underestimated issue and demonstrate the value of cardiopulmonary exercise testing (CPX) for diagnosis and exercise prescription in this population.

## Case presentation

Two male fire survivors (Case A, a 33-yr-old and Case B, a 40-yr-old, respectively) were sent to hospital because of smoke exposure in a burning building, complaining of hoarseness, sore throat, and cough. They denied any history of cigarette smoking or medical disease. Case A presented with fever (38.3 °C), mild tachycardia (122 bpm), and elevated SBP/DBP (172/106 mmHg). Case B was afebrile (37.2 °C) with a normal heart rate (HR, 91 bpm) and systolic/diastolic blood pressure (SBP/DBP, 122/62 mmHg). Physical examinations revealed clear consciousness, normal respiratory rate (20 respirations per minute), and clear breathing sounds. No stridor, signs of burn injury, and respiratory distress were noted in either case.

Under supplemental oxygen (O_2_) via a mask (O_2_ flow: 10 L/min), arterial blood analysis showed respiratory alkalosis in Case A and Case B (pH: 7.479 and 7.506, the partial pressure of O_2_: 249.5 mmHg and 389.4 mmHg, the partial pressure of carbon dioxide: 26.7 mmHg and 27.2 mmHg, bicarbonate levels: 19.4 mmol/L and 21.0 mmol/L, peripheral O_2_ saturation: 99.2% and 99.1%, respectively). Initial serum analysis revealed elevated carboxyhemoglobin (COHb) levels (8.2% in Case A and 14.8% in Case B). Chest radiography revealed increased lung marking, and a resting electrocardiogram (ECG) showed sinus tachycardia in Case A and sinus rhythm in Case B. Fiberoptic bronchoscopies were arranged for the patients to assess smoke inhalation injury, which revealed smoke-soaked nasal discharge and no upper airway obstruction. They were admitted for further management and observation following smoke inhalation injury with CO intoxication.

They both received bronchodilator and glucocorticoid therapies for airway edema. High-flow O_2_ therapy via non-rebreathing masks and hyperbaric O_2_ therapy were administered for CO intoxication. Case A became afebrile, with a reduction in HR and SBP/DBP on the next day. Follow-up COHb levels decreased to 3.9% and 0.8% in cases A and B, respectively. There was no chest discomfort or ischemic evidence in serial ECG during hospitalization in either case. However, in Case B, the cardiac enzyme level was elevated initially and decreased on the next day (high-sensitive cardiac troponin T level decreased from 13.5 ng/L to 4.0 ng/L). The hoarseness, sore throat, and cough improved after 4-day of hospitalization.

**Fig. 1:**
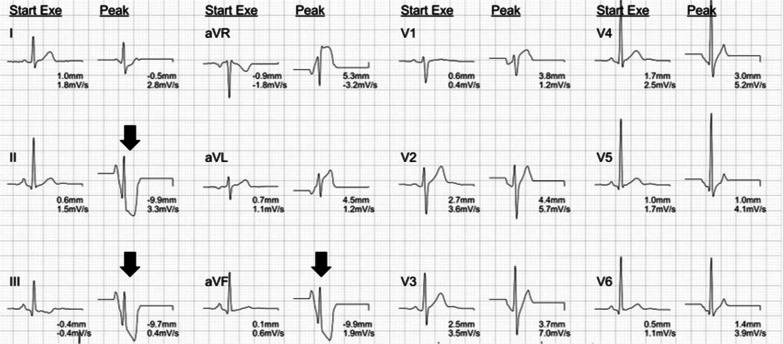
Exercise electrocardiogram of Case A showed a marked downsloping ST-segment depression (9.7–9.9 mm) in lead II, III, and aVF at peak exercise (arrow), which indicated exercise-induced myocardial ischemia

Both patients experienced exercise intolerance after discharge while participating in physical activities of the previous level. Early fatigue was noted before reaching the previous exercise intensity. After a general assessment, including physical examination, blood testing, and chest radiography, they were referred to physiatrists by pulmonologists for further investigation to evaluate exercise intolerance. Approximately 2-week after discharge, spirometry tests showed normal pulmonary function. CPX performed on a treadmill using the Bruce protocol [3], revealed peak oxygen consumption (VO_2_ peak) of 38.7 mL kg^− 1^ Min^− 1^ and 37.6 mL kg^− 1^ min^− 1^, respectively; however, there was a slight decrease in the percentage of predicted VO_2_ peak (90.63% and 94.47%). Peak HR, breathing reserve, and minute ventilation/carbon dioxide production relationship were within normal limits. No chest discomfort was reported during testing. However, at peak exercise, they presented with a significantly downsloping ST-segment depression in lead II, III, and aVF, which indicated exercise-induced myocardial ischemia (Fig. 1 and Additional file 1: Supplementary Fig. 3). CPX also demonstrated the presence of disproportionately increasing HR with elevated BP and O_2_-pulse failure from stage 3 of exercise to peak exercise (Additional file 1: Supplementary Fig. 1 and Supplementary Fig. 4). Selective parameters of the spirometry test and abnormalities of CPX are further demonstrated in Table 1 and Additional file 1: Fig. Supplementary Fig. 2 and Supplementary Fig. 5.

**Table 1:** Characteristics and principal variables in cardiopulmonary test

Variables^b^		Case A	Case B
Age (year)		33	40
Sex		Male	Male
Body height (cm) / weight (kg)		173 / 62	175 / 85
Initial COHb (%)	[normal range]	8.2 [0.0–3.0]	14.8 [0.0–3.0]
FEV_1_ (L)	[% predicted value]	3.68 [95]	3.54 [93]
FEV_1_/FVC	[% predicted value]	88.31 [105]	74.98 [91]
^a^Heart rate (bpm)	[% predicted value]	188 [100]	181 [101%]
VO_2 peak_ (mL· kg^− 1^ · min^− 1^)	[% predicted value]	38.7 [90.63]	37.6 [94.47]
VO_2 AT_ (mL· kg^− 1^ · min^− 1^)	[%VO_2 peak_]	22.3 [58]	19.3 [52]
^a^O_2_ pulse (mL· beat^− 1^)	[% predicted value]	14 [100]	18 [94.73]
Breathing reserve (%)	[normal range]	48.4 [> 10]	40 [> 10]
V_E_/VCO_2 nadir_	[normal range]	28 [25.0 ± 2.7]	25 [25.0 ± 2.7]
V_E_/VCO_2_ slope	[normal range]	24.4 [23.9 ± 3.1]	23.6 [23.9 ± 3.1]
SpO_2 nadir_ (%)	[maximal exercise]	95 [97]	94 [94]
OUES (mL/min/log(L/min))	[% predicted value]	2289 [98.72]	3579 [117.34]
^a^Respiratory exchange ratio		1.17	1.13
Exercise time (min’sec”)		10’14”	9’58”
^a^Systolic blood pressure/ Diastolic blood pressure (mmHg)	[at stage 3]	161/75 [166/71]	151/69 [140/54]

COHb, carboxyhemoglobin; FEV_1_, forced expiratory volume in 1 s; FVC, forced vital capacity; OUES, oxygen uptake efficiency slope; V_E_/VCO_2 nadir_, the nadir of the ventilatory equivalent for carbon dioxide; V_E_/VCO_2_ slope, the slope of minute ventilation as a function of carbon dioxide production in the range of exercise below the ventilatory compensation point; VO_2 AT_, oxygen consumption at the anaerobic threshold

^a^Indicate peak value obtained during peak exercise.

^b^Normal value and predicted value are based on the CPX laboratory findings in this study and Wasserman & Whipp’s Principles.^8^

The tests were stopped as the HR peak was attained with progressive ECG signs of myocardial ischemia with a duration of 10 min 14 s and 9 min 58 s, respectively. Finally, transient myocardial ischemic changes on ECG returned to normal within 2 min of recovery. For exercise suggestion, HR method were instructed for guidance the intensity zone, as their myocardial ischemic threshold referred to the HR once the ST-segment depression was 1.5 mm (0.15 mV) in CPX (approximately 160 bpm). They were instructed to exercise with precaution in the community setting with slow progression. No adverse cardiopulmonary events were reported. In addition, exercise intolerance improved subjectively during the 6-month follow-up and returned to baseline.

## Discussion and conclusions

To the best of our knowledge, this is the first case report to describe exercise-induced myocardial ischemia and exercise intolerance in fire survivors following CO intoxication and smoke inhalation injury, even after comprehensive supportive treatment [1, 4].

CO has a higher affinity for hemoglobin than for O_2_. Once inhaled, CO competes with O_2_ to form COHb in the red blood cells, and binds to myoglobin to form carboxymyoglobin through circulation [5]. In the mitochondria, it also competes against O_2_ binding to cytochrome oxidase and inhibits the electron transport chain, which causes intoxication-related tissue hypoxia. Generally, COHb could be up to 5% in normal people and up to 9% in smokers [6]. Herein, both non-smoker patients with initial COHb levels of 8.2% and 14.8% indicated CO intoxication. Beyond unspecific symptoms presented in CO intoxication, cardiac toxicity is common due to the high blood flow and oxygen demand of the myocardium.

The interpretation of CPX for exercise intolerance in present cases was of interest. Pulmonary related exercise limitations due to smoke inhalation injury were excluded based on normal findings in pulmonary function tests, peripheral O_2_ saturation, and ventilation-perfusion relationships (reflected by normal VE/VCO_2_ slope and VE/VCO_2_ nadir). Early mild cardiovascular diseases was considered, which was supported by an essentially normal VO_2_ peak and abnormality in the exercise ECG [7], and the most common important diagnosis to be identified is ischemic heart disease [7, 8]. The standard ECG criterion for exercise-induced myocardial ischemia in ST-segment depression is horizontal or downsloping ST-segment depression ≥ 0.10 mV (1 mm) based on 80 msec[7]. Downsloping ST-segment depressions are more specific because upsloping ST-segment depression at peak exercise might be found in 10–20% of normal people [9]. O_2_ pulse, a product of stroke volume and arteriovenous O_2_ difference, rises normally with a gradually decreasing rate of elevation to the predicted normal value in healthy people. Herein, the diagnosis of exercise-induced myocardial ischemia was further supported by disproportionately increasing HR with elevated BP and O_2_ pulse failure from stage 3 of exercise to peak exercise [7].

In addition to severe myocardial infarction, CO intoxication may induce variant angina and cause the “stunned myocardium-like syndrome” with completely normal coronary angiograms and focal hypokinesia [10, 11]. Based on the recent history of CO intoxication and CPX findings in these cases, exercise intolerance was likely to be exercise-induced myocardial ischemia, which may be caused by myocardial or coronary arterial dysfunction induced by CO intoxication.

Cardiac rehabilitation has been shown beneficial in increasing quality of life and reducing mortality and morbidity in patients with cardiovascular diseases [12]. Setting exercise intensity is a critical part of exercise prescription in ischemic heart disease. According to the guidelines of the American College of Sports Medicine (ACSM), if the ischemic threshold is identifiable (i.e., angina and/or ≥ 1 mm ischemic ST-segment depression on exercise ECG), the upper limit of the HR should be set at a minimum of 10 bpm below the HR at the ischemic threshold [13]. Accordingly, the exercise suggestion and precaution in our cases were set at a target HR of approximately 145 bpm with slow progression, which was based on the guidelines of the ACSM and modified by an experienced senior physiatrist [13].

Younger people may suffer from a myocardial injury caused by moderate-to-severe CO intoxication despite appearing to be a low-risk population from a cardiovascular standpoint [14]. A nationwide database cohort study in Taiwan reported that patients with CO intoxication had an increased risk of myocardial infarction (incidence rate ratio of 1.45) with more prominence in young age (< 34 year), female sex, and liver disease, and occurred only in the first month of follow-up [15].

Diagnostic tools, including cardiac markers, brain natriuretic peptide, ECG, echocardiogram, scintigraphy, and coronary angiography, are used to evaluate myocardial injury in patients with CO intoxication. However, current diagnostic algorithm is often difficult to detect the cardiac toxicity and related exercise limitations [2]. CPX, combined maximal or symptom-limited progressive intensity exercise with subjective symptoms, serial ECG, hemodynamics, peripheral O_2_ saturation, and breath-by-breath ventilatory expired gas analysis, can provide quantified data of cardiorespiratory fitness and diagnostic values for exercise limitation [16, 17], and can be used for myocardial injury screening, especially for those presenting with functional complaints such as exercise intolerance.

.

During the 6-month follow-up in our cases, no adverse cardiopulmonary events were reported; however, outcome assessment was limited methodically. Due to both patients refusing follow-up CPX after receiving cardiac rehabilitations with improved symptoms. The effectiveness of cardiac rehabilitation remained undetermined by objective evaluation. Currently, there is no consensus from evidences to guide diagnosis and management of exercise-induced myocardial ischemia after CO intoxication and smoke inhalation injury. Due to a significant increase in the risk of long-term mortality in patients with CO intoxication, a screening protocol for exercise intolerance with CPX assistance may be a potential method to match the need for early follow-up and secondary prevention [18]. Further large-scale studies are needed, focusing on the early assessment of exercise intolerance and comprehensive exercise prescription in these populations.

In conclusion, exercise intolerance after carbon monoxide intoxication and smoke inhalation injury in low cardiovascular risk population may be underestimated. More attention should be paid to fire survivors, especially after CO intoxication, to improve short-term and long-term outcomes. CPX plays a role in the diagnosis and guidance of treatment of exercise-induced myocardial ischemia.

## Supplementary Information

**Additional file 1.** Case presentation.

## Data Availability

The data presented in this study are available on request from the corresponding author. The data are not publicly available owing to privacy.

## Abbreviations

CPX: Cardiopulmonary exercise testing
COHb: Carboxyhemoglobin
ECG: Electrocardiogram
VO_2_ peak: Peak oxygen consumption
