# Supplementary material for: Exercise-induced myocardial ischemia presenting as exercise intolerance after carbon monoxide intoxication and smoke inhalation Injury: case report
Source: BMC Cardiovasc Disord. 2022 Dec 27;22:570. doi: 10.1186/s12872-022-03019-4 (PMC9795777; doi:10.1186/s12872-022-03019-4)

**Exercise-Induced Myocardial Ischemia Presenting as Exercise Intolerance After Carbon Monoxide Intoxication and Smoke Inhalation Injury: Case Report**

**Supplemental Material:**

Supplemental Figure 1-5

**Supplemental Figure 1** Heart rate and oxygen pulse plotted as functions of time and stages of cardiopulmonary exercise testing (Case A)


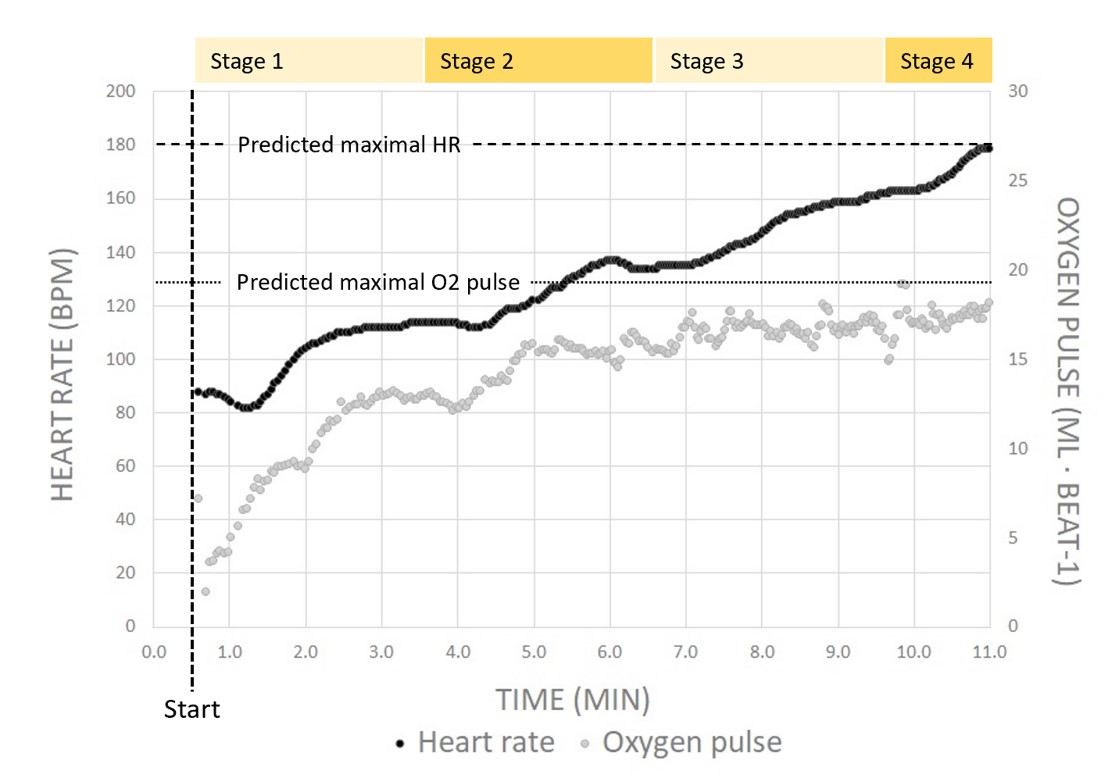


**Supplemental Figure 2** Selective parameters during cardiopulmonary exercise testing of Case A. VO_2_, oxygen consumption (L min^–1^); VCO_2_, carbon dioxide production (L min^–1^); VE, minute ventilation; VE/VO_2_, minute ventilation divided by oxygen consumption; VE/VCO_2_, minute ventilation divided by carbon dioxide production; RER, respiratory exchange ratio

**
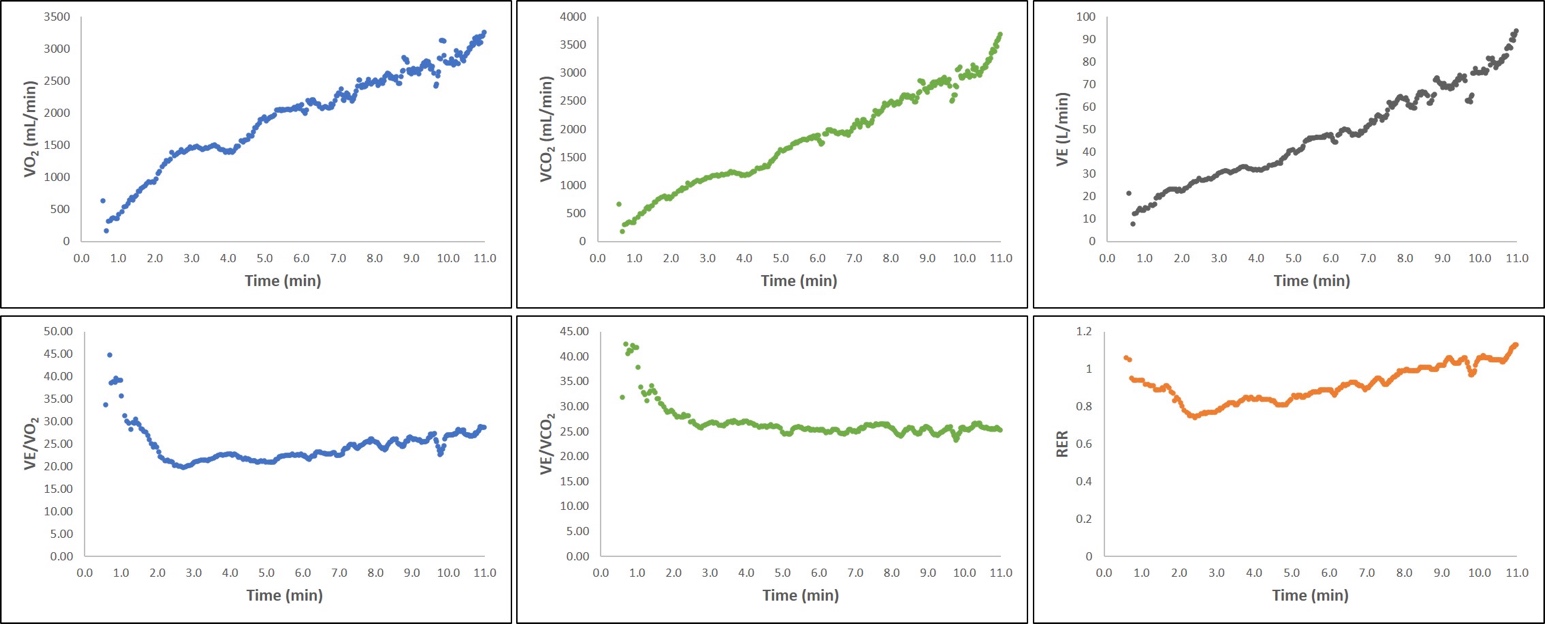
**

**Supplemental Figure 3** Exercise electrocardiogram of Case B, showed a marked downsloping ST-segment depression in lead II, III, and aVF at peak exercise (arrow)


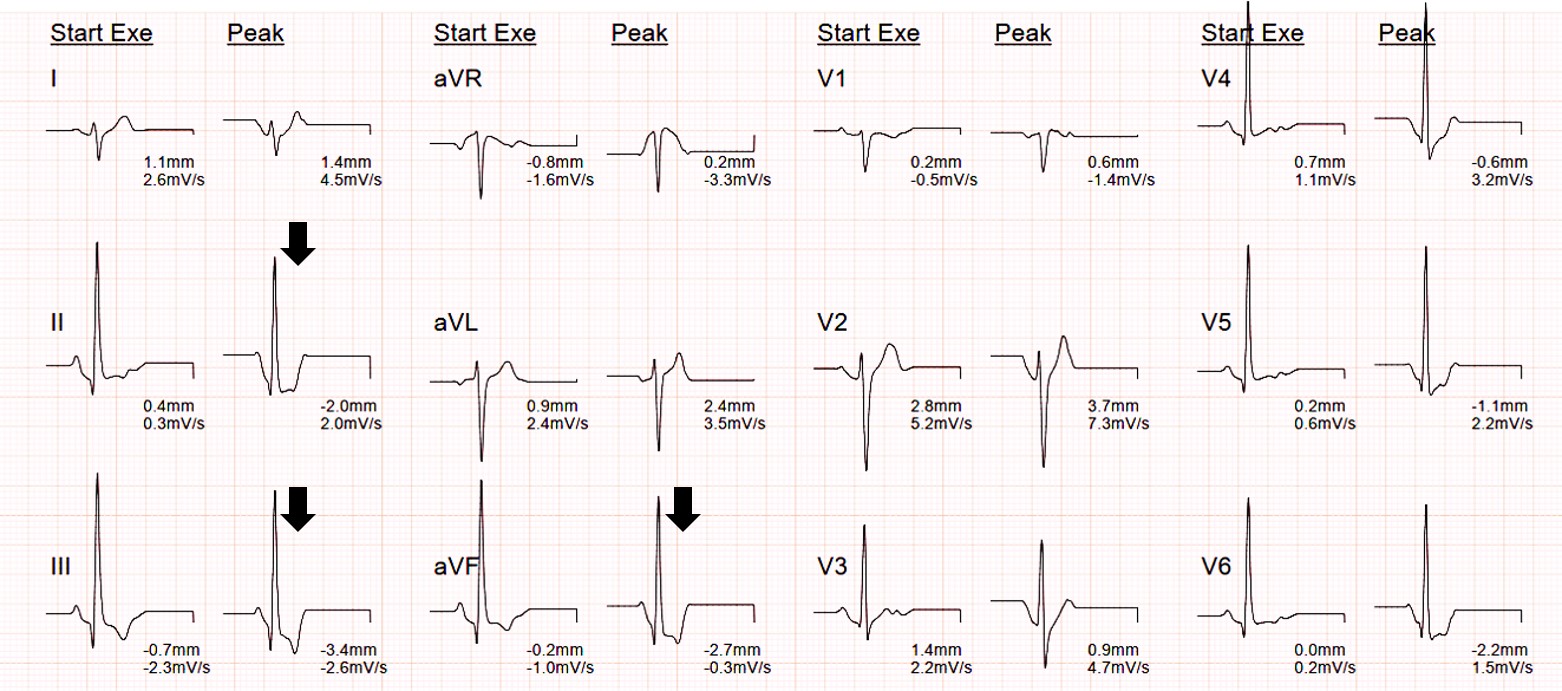


**Supplemental Figure 4** Heart rate and oxygen pulse plotted as functions of time and stages of cardiopulmonary exercise testing (Case B)


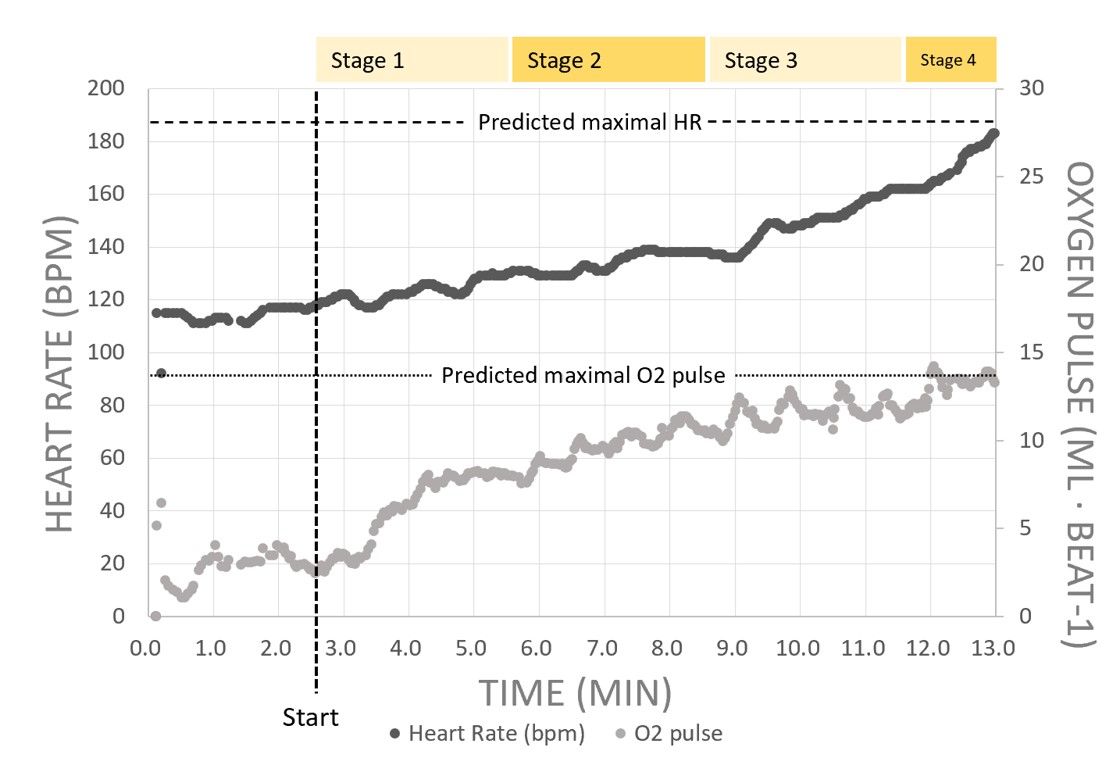


**Supplemental Figure 5** Selective parameters during cardiopulmonary exercise testing of Case B. VO_2_, oxygen consumption (L min^–1^); VCO_2_, carbon dioxide production (L min^–1^); VE, minute ventilation; VE/VO_2_, minute ventilation divided by oxygen consumption; VE/VCO_2_, minute ventilation divided by carbon dioxide production; RER, respiratory exchange ratio


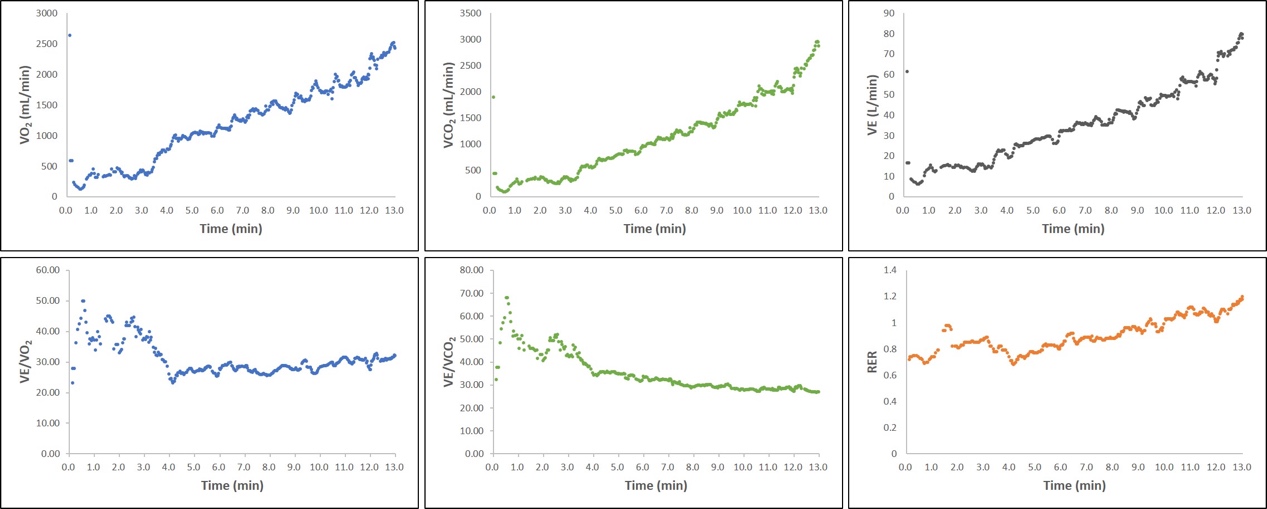

Supplement: Supplementary file 1 — Additional file 1. Case presentation. [file 12872_2022_3019_MOESM1_ESM.docx]
